# Bacterial Dynamics in the Accessory Nidamental Gland of *Sepioteuthis lessoniana* throughout Maturation

**DOI:** 10.1264/jsme2.ME21030

**Published:** 2021-10-02

**Authors:** Shan-Hua Yang, Chi Chen, Yunli Eric Hsieh, Sung-Yin Yang, Hau-Wen Li, Tzu-Yun Ching, Chia-Hui Wang, Ching-Fong Chang, Sen-Lin Tang, Guan-Chung Wu

**Affiliations:** 1 Institute of Fisheries Science, National Taiwan University, Taipei, Taiwan; 2 Doctoral Degree Program in Marine Biotechnology, National Taiwan Ocean University, Keelung, Taiwan; 3 Doctoral Degree Program in Marine Biotechnology, Academia Sinica, Taipei, Taiwan; 4 Shimoda Marine Research Center, University of Tsukuba, Shimoda, Japan; 5 National Museum of Marine Biology and Aquarium, Pingtung, Taiwan; 6 Department of Aquaculture, National Taiwan Ocean University, Keelung, Taiwan; 7 Department of Environmental Biology and Fisheries Science, National Taiwan Ocean University, Keelung, Taiwan; 8 Center of Excellence for the Oceans, National Taiwan Ocean University, Keelung, Taiwan; 9 Biodiversity Research Center, Academia Sinica, Taipei, Taiwan

**Keywords:** *Sepioteuthis lessoniana*, accessory nidamental gland, maturity, microbial dynamics

## Abstract

The accessory nidamental gland (ANG) is part of the reproduction organ in the majority of female cephalopods, including the bigfin reef squid *Sepioteuthis lessoniana*, an economically important fishery product. Microbes in *Alphaproteobacteria*, *Gammaproteobacteria*, and *Verrucomicrobia* have been suggested to play a role in the maturation of the *S. lessoniana* ANG and are responsible for its color. However, the bacterial composition and dynamics of the different maturation stages of the ANG remain unclear. In the present study, we surveyed ANG-associated bacterial dynamics in wild-caught *S. lessoniana* at various developmental stages in different populations over 3 years. The results obtained showed that the ANG bacterial community shifted gradually and decreased in diversity throughout maturation. *Verrucomicrobia* occupied the ANG during the early stages in large numbers, and was replaced by *Bacteroidia*, *Alphaproteobacteria*, and *Gammaproteobacteria* in the later stages. *Flavobacteriales* and *Alphaproteobacteria* both appeared to contribute to pigmentation, while *Bacteroidia*, *Alphaproteobacteria*, and *Gammaproteobacteria* may be involved in enriching the heme biosynthesis pathway in the ANG with the maturation of *S. lessoniana*. The present results provide an open question of whether *S. lessoniana* actively selects the bacterial community in the ANG to adjust to its surrounding environment.

The bigfin reef squid *Sepioteuthis lessoniana* is a widespread loliginid squid species in the Indo-Pacific Ocean region, and represents one of the most economically important fishery products in Taiwan. This species is closely associated with coral reef habitats, and lays jelly-like egg strings in seagrasses, sea fans, coral rubble, and reefs (Norman, 2003). Between April and October in Taiwan, *S. lessoniana* swims in groups to the shore for spawning. The population structure of *S. lessoniana* in Taiwan has been investigated using life history parameters, statolith geochemical signatures, and genetic analyses (Ching *et al.*, 2019; Chiang *et al.*, 2020), and one predominant taxon was found in the water of northeastern Taiwan.

*S. lessoniana* trading still mainly relies on capture fishing; however, culturing techniques have also been established due to the high demand for this species. Aquaculture breeding techniques for this species need to be improved in order to reach market volumes. Current bottlenecks around the aquaculture production of cephalopods may be due to a lack of detailed information on the processes of maturation, reproduction, and hatching in natural environments and under cultivation conditions (reviewed in O’Brien *et al.*, 2018), with limited information being available on microbial communities.

The accessory nidamental gland (ANG) is part of the reproduction organ that is present in the majority of female cephalopods. *Alphaproteobacteria*, *Gammaproteobacteria*, and *Verrucomicrobia* are common microbial taxa that associate with different species of cephalopod ANG (Pichon *et al.*, 2005; Collins *et al.*, 2012; Kerwin and Nyholm, 2017). Since juvenile cephalopods do not have a developed ANG, the microbial community in the ANG is considered to undergo horizontal transfer from the environment (Kaufman *et al.*, 1998). The microbial community composition may be related to ANG maturity. During maturation, the ANG turns from white to an orange color that originates from the carotenoids produced by the microbiome (Lum-Kong and Hastings, 1992). One of the functions of the ANG is to provide bacteria in the egg jelly coat (JC) as a defense mechanism against microorganisms, such as those in the ANG of the Hawaiian bobtail squid, *Euprymna scolopes* (Collins *et al.*, 2012; Kerwin and Nyholm, 2017).

Female *S. lessoniana* also have an ANG; however, information on the microbial community associated with this ANG is limited. Li *et al.* (2019) hypothesized that as bacteria move from marine environments into the ANG, the transferrin (TF)-like protein of the host may play an essential role in microbial selection. During the maturation process, the ANG gradually transfers bacteria from the outer epithelial cell layer, invaginating bacteria into the ANG and forming tubule structures for bacterial colonies (Li *et al.*, 2019). The TF-like protein, which is rich in the epithelial cell layer and the early stages of the ANG (Li *et al.*, 2019), may be the key for initial bacterial selection. However, the microbial composition in different ANG maturation stages and the dynamic composition throughout the maturation process remain unknown. Therefore, the present study surveyed ANG-associated bacterial dynamics in *S. lessoniana* at various developmental stages in different populations over 3 years. This is the first survey of the ANG of wild-caught populations. Our three-year field sampling of different stages of ANG maturation tracked resident bacterial communities using NGS amplicon sequencing to provide more detailed insights into the core organ-specific microbiome in certain ANG development stages.

## Materials and Methods

### Squid collection

Bigfin reef squids were purchased from a fisherman on Hoping Island, Keelung City, Taiwan. Squids were collected at night by hand jigging on a boat in the northeast coast of Taiwan (Fig. S1), and were maintained using a fresh seawater system on the boat. They were transferred to a seawater tank (2.5 tons FRP tank) with a circulating seawater system the next morning until tissue sampling (less than 2 hrs). The ANG of squids were collected after 6–12 h of fishing on the boat, which ensured that additional bacteria were not acquired in captivity. Squids were anesthetized in seawater containing 5% ethanol at room temperature and then euthanized by cutting off the head. All procedures and investigations were approved by the National Taiwan Ocean University Institutional Animal Care and Use Committee and were performed in accordance with standard guidelines.

**Fig. 1.**
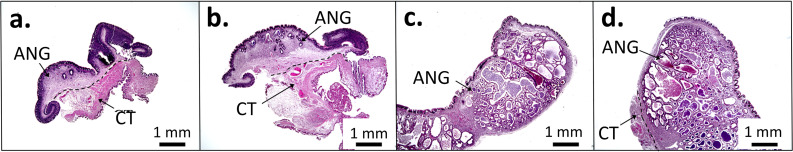
Histological analysis to indicate different stages of the accessory nidamental gland of *Sepioteuthis lessoniana*. (a) to (d) indicate stages 1 to 4, respectively. ANG, accessory nidamental gland; CT, connective tissue. The dashed line denotes the barrier between the ANG and CT.

To elucidate the relationship between the microbiota of the ANG and the female reproductive cycle, different ANG at various stages were collected for histological and amplicon analyses. Based on the techniques used for squid collection (hand jigging on a boat), it is difficult to collect seawater samples in natural habitats. Three batches of female squids were used in the present study between 2015 and 2018: autumn 2015 to spring 2016 (batch 1), autumn 2016 to spring 2017 (batch 2), and autumn 2017 to spring 2018 (batch 3) (Table 1). The ANG stage was assessed based on morphological characteristics in accordance with our previous study (Li *et al.*, 2019). Four developmental stages of the ANG were recognized in the present study (Fig. 1): a colorless ANG without any bacterial aggregates in juvenile squids (stage 1), a colorless ANG with bacterial colonies in immature squids (stage 2), a white/light-orange ANG with bacterial colonies in maturing squids (stage 3), and a pigmented ANG with a large number of bacterial colonies in mature squids (stage 4).

### Taxa identification

More than three potential bigfin reef squid taxa have been observed in its distribution area: *Sepioteuthis* sp. 1, sp. 2, sp. 2A, and sp. 3 (Cheng *et al.*, 2014; Ching *et al.*, 2021). The dominant taxon around northeastern Taiwan is *Sepioteuthis* sp. 1 (Cheng *et al.*, 2014; Ching *et al.*, 2021). To avoid differences in ANG bacterial communities due to lineage-specific selection, we used only one lineage—*Sepioteuthis* sp. 1—as the target for the present study. Total DNA was extracted using the QIAamp DNA Mini Kit (Qiagen) from the frozen muscle tissue of each bigfin reef squid, based on the manufacturer’s protocol. Multiplex cytochrome oxidase c subunit I (COI) haplotype-specific PCR (MHS-PCR) was performed to identify the potential lineages of squids using the following primers: AF-5′-TCTCATGCTGGACCTTCAGTA-3′; AR-5′-TGCTCCTGCTAAAACAGGAAG-3′; BF-5′-ATTGGGGGTTTTGGTAACTGG-3′; BR-5′-GATGCTAAAAGGAGTGTGAGG-3′; C1F-5′-TTAGTTGGTACCTCACTAAGG-3′; C1R-5′-CTCTTTCAACTGCTGAGGAC-3′; C2F-5′-TTAGTTGGTACCTCACTAAGG-3′; C2R-5′-GTTGATATAGAATAGGGTCTCCC-3′. These haplotype-specific primers were designed based on bigfin reef squid samples collected around Taiwan (Hsiao *et al.*, 2016; Shen *et al.*, 2016). PCR was performed using a PCR Thermal Cycler (Gene Atlas), each with 9.8‍ ‍μL of reaction solution containing 0.2‍ ‍ng template DNA, 0.3‍ ‍μM of each primer, 0.2‍ ‍mM dNTP mixture, 10× PCR buffer (20‍ ‍mM Tris–HCl pH 8.0, 15‍ ‍mM KCl, and 15‍ ‍mM MgCl_2_), and 5‍ ‍U μL^–1^ DNA polymerase (Thermo Scientific). Reaction conditions were as follows: initial denaturation at 94°C for 5‍ ‍min; 35 cycles of denaturation at 94°C for 45‍ ‍s, annealing at 60°C for 1‍ ‍min, and extension at 72°C for 1‍ ‍min; and a final extension at 72°C for 10‍ ‍min. Amplified products were confirmed using 1.5% agarose gel electrophoresis, and we observed a band corresponding to one of the lineages. The bands for *Sepioteuthis* sp. 1, sp. 2, sp. 2A, and sp. 3 were 207, 122, 281, and 680 bp, respectively.

### Total DNA extraction, PCR, barcoding PCR, and sequencing

The ANG stages of all squids were assessed based on morphological characteristics (listed in Table 1). The microbiota of 36 female squids at different developmental stages were analyzed. ANG were homogenized in Trizol reagent (Invitrogen). This nucleoprotein complex was mixed with chloroform to separate RNA, DNA, and proteins in the upper aqueous phase, lower phenol-chloroform phase, and interphase, respectively. Total DNA was precipitated from the phenol-chloroform phase using 100% ethanol. Isolated total DNA was used for an amplicon analysis. The equipment used was sterilized and free of RNase to prevent contamination during the experiment.

PCR was performed using two bacterial universal primers—361F (5′-CCTACGGGNGGCWGCA-3′) and 806R (5′-GACTACHVGGGTATCTAATCC-3′)—which were designed for the bacterial V3V4 high variable region of the 16S ribosomal RNA gene (Nossa *et al.*, 2010). Thirty cycles of PCR were performed as follows: an initial step of 94°C for 3‍ ‍min, 94°C for 15‍ ‍s, 55°C for 15‍ ‍s, and 68°C for 1‍ ‍min, with 2‍ ‍min at 72°C as the final extension after the last cycle. PCR amplicons were checked by DNA agarose gel electrophoresis with a 1.5% agarose gel and 0.5× TAE buffer. PCR-amplified 350–450-bp fragments were purified using the QIAquick Gel Extraction Kit (Qiagen). The quality of DNA was assessed using a NanoDrop^TM^ 1000 spectrophotometer (Thermo Scientific). DNA library construction and Illumina sequencing were performed by Genomics. TruSeq DNA Sample Preparation Kits (Illumina) were used for library construction, and prepared libraries were quantified using a GeneRead Library Quant Kit (Qiagen) before loading them into the NGS sequencer, MiSeq (Illumina). Five hundred nanograms of each purified PCR product were subjected to Illumina-based high-throughput sequencing.

### Bioinformatic and statistical analyses

An amplicon analysis was performed as described by Tandon *et al.* (2018). Amplicons were quality checked (average phred score ≥27) and poor quality reads were trimmed. The trimming of merged sequences was conducted by mothur software with a minimum length=440 bp and maximum length=466 bp (Schloss *et al.*, 2009). After trimming, chimeric reads were detected by UCHIME (http://drive5.com/uchime) (Edgar *et al.*, 2011). In the operational taxonomic unit (OTU) analysis, quality-filtered reads were pooled together and analyzed with the UPARSE pipeline (Edgar, 2013) without the chimera removal step. In UPARSE, de-replication was performed with the options “–derepfulllength” and “–minsize 2” and OTUs were generated at 97% identity. Each OTU was searched (with global alignment) against the Silva132 database to find the corresponding taxonomy of the best hit using USEARCH (Edgar, 2013). Alignment was calculated using MOTHUR software to define OTUs with a 20% cut-off value for sequence dissimilarity. Defined OTUs were used to estimate the Shannon-Weaver diversity index, Chao1 estimator, and the Simpson index as well as to construct rarefaction curves and calculate the evenness and richness of the bacterial community. Unclassified OTUs were manually searched in NCBI using BLASTn. The relative abundance of each classified bacterial class in individual samples was incorporated into a matrix. A total of 2,310,443 qualified reads were obtained from the 36 samples and 594 OTUs were generated. After the primer was removed, sequencing reads were deposited into the NCBI Sequence Read Archive database under accession number PRJNA666041.

### Biodiversity and statistical analyses

Results were presented in hierarchical clustering (CLUSTER) and non-Metric multi-Dimensional Scaling (nMDS) using R (PRIMER-E) to analyze the relationships between bacterial communities among samples.

### Functional prediction

PICRUst2 (https://github.com/picrust/picrust2) was used to predict bacterial functions based on the bacterial OTUs of the ANG. The total OTU table, 191 core OTUs, *Alphaproteobacteria* OTUs, *Gammaproteobacteria* OTUs, and *Bacteroidia* OTUs were used to predict KEGG Orthology (KO) relative abundance. A metabolic module enrichment analysis was performed with a functional sets enrichment analysis (FSEA) as described by Liu *et al.* (2020). The ‘FSEA’ function in the MARco R package based on Liu *et al.* (2020) was applied (https://github.com/poyuliu/MARco). The metabolic module enrichment analysis was conducted with the R package gage (testing using the ‘gage’ function to assess metabolic module enrichment by comparing equal gene abundance distribution within groups and by comparing mean gene abundance among groups; significant modules were identified from one-tailed tests for up-regulation). The *P* values from multiple tests were adjusted with a false discovery rate (FDR), with a *P* value-adjusting function embedded in the gage package; the significance of enrichment analyses was defined by FDR q-value <0.05. Enrichment scores were calculated according to the gene-set enrichment analysis (GSEA) algorithm of DAVID bioinformatics resources.

## Results

The rarefaction curve showed that all samples plateaued (Fig. S2). The range of species richness differed each year; however, richness always slightly declined from the earlier stage (stages 1 and 2) to the later stage of the ANG. Alpha diversity indicated no significant differences among the three years; therefore, bacterial richness/evenness was similar across the years (Fig. S3). On the other hand, bacterial compositions at the OTU level significantly differed among the years (ANOSIM, R^2^=0.286, *P*=0.01) (Fig. S4), although those in 2016 and 2017 were similar. This result implied that environmental conditions or bacterial diversity in seawater influences the basic bacterial constitution of the ANG of *S. lessoniana*.

**Fig. 2.**
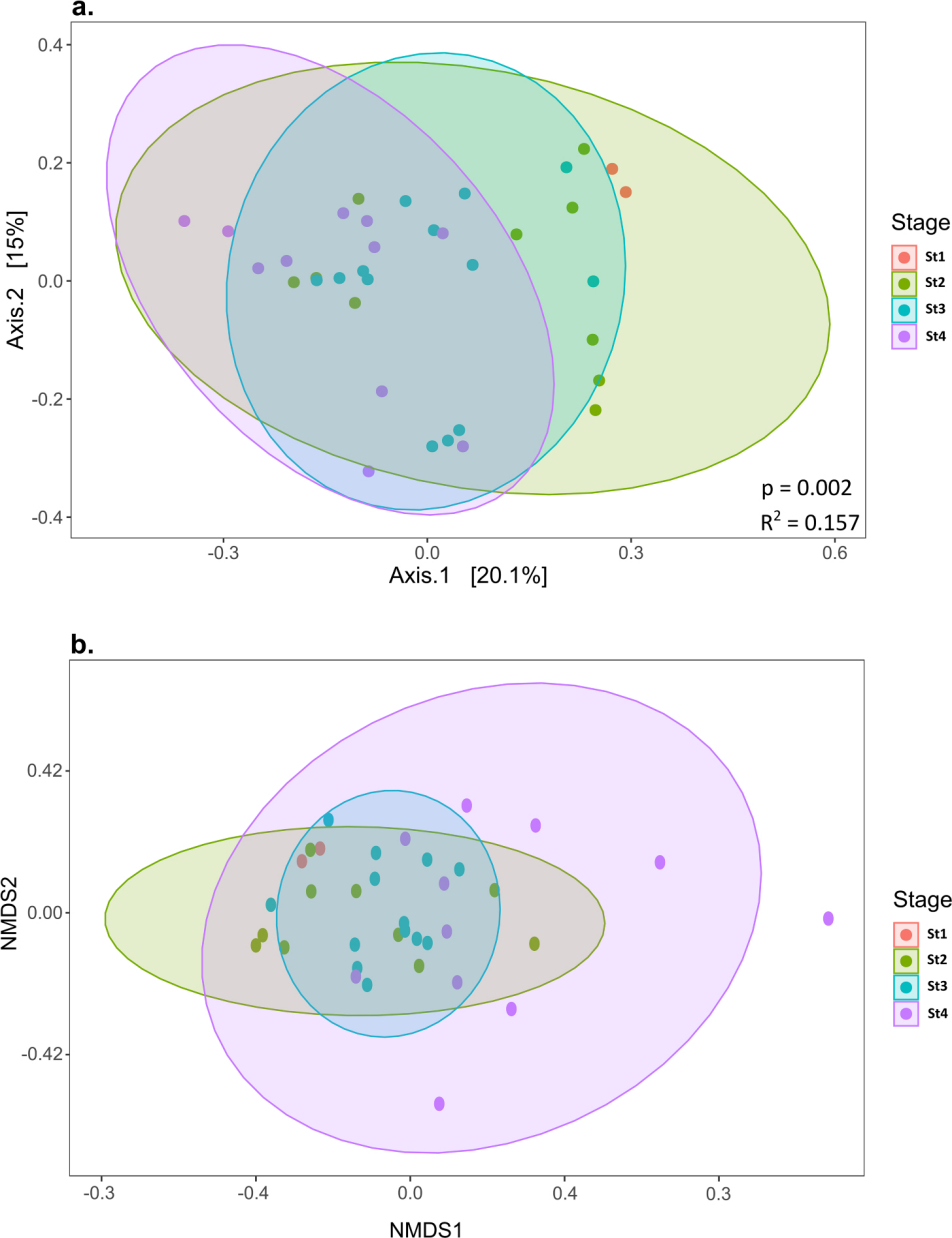
Beta diversity of bacterial OTUs from different stages. (a) A principal coordinate analysis (PCoA) of bacterial OTUs from different stages. (b) Non-metric multidimensional scaling (nMDS) of bacterial genera from different stages in present/absent transformation.

In comparisons of diversity indices across sampling stages (Fig. S5), Chao 1 and Simpson showed that stages 1 and 2 had higher richness and evenness than the late stage. PCoA (Fig. 2a) revealed that the bacterial composition of each stage showed a change with a linear pattern along the first axis, whereas the majority overlapped. Stage 1 samples were separated from stage 4 samples, indicating bacterial succession in the ANG during maturation. In addition, the nMDS of the bacterial genus composition showed a similar pattern among stage 1, 2, and 3 individuals, but a markedly different pattern among stage 4 individuals (Fig. 2b).

**Fig. 3.**
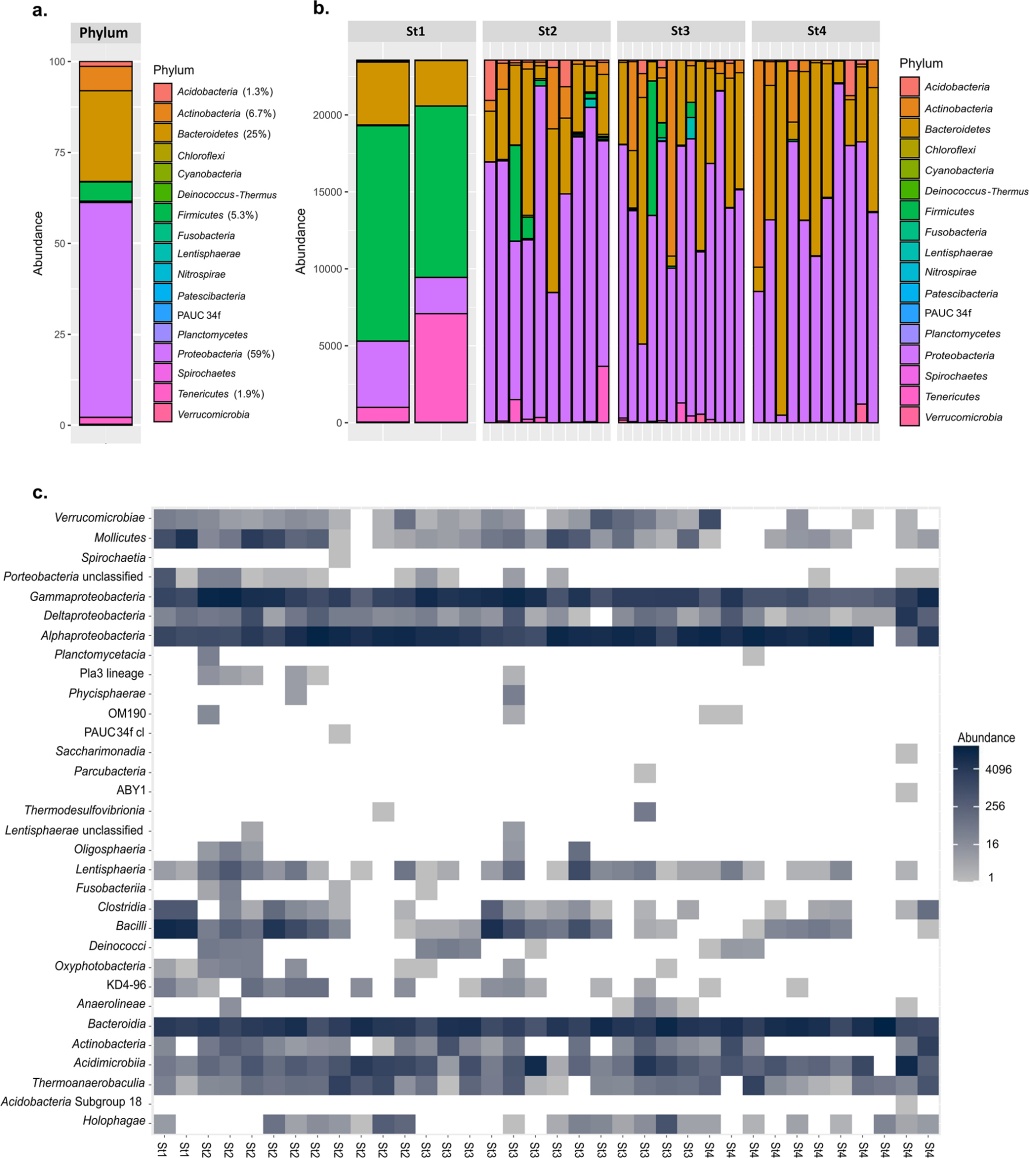
*Sepioteuthis lessoniana* ANG-associated bacterial composition. (a) Bacterial composition at the phylum level. (b) Bacterial composition at the phylum level for each sample. (c) *Sepioteuthis lessoniana* ANG-associated bacterial composition at the class level.

Regarding bacterial compositions, the *S. lessoniana* ANG mainly comprised *Proteobacteria* (59%), *Bacteroidetes* (25%), *Actinobacteria* (6.7%), and *Firmicutes* (5.3%) (Fig. 3a). The phyla in stage 1 markedly differed from those in other stages. In stage 1, *Firmicutes* was the dominant phylum, while the abundance of *Proteobacteria* and *Bacteroidetes* increased in later stages (Fig. 3b). In all stages, the dominant class were *Gammaproteobacteria*, *Alphaproteobacteria*, and *Bacteroidia*, and the abundance of *Bacteroidia* slightly increased in stages 3 and 4. Some classes decreased throughout the maturation stages: *Verrucomicrobiae*, *Mollicute*, *Clostridia*, *Bacilli*, *Oxyphotobacteria*, KD4-96 (*Chloroflexi*), and *Actinobacteria* (Fig. 3c).

**Fig. 4.**
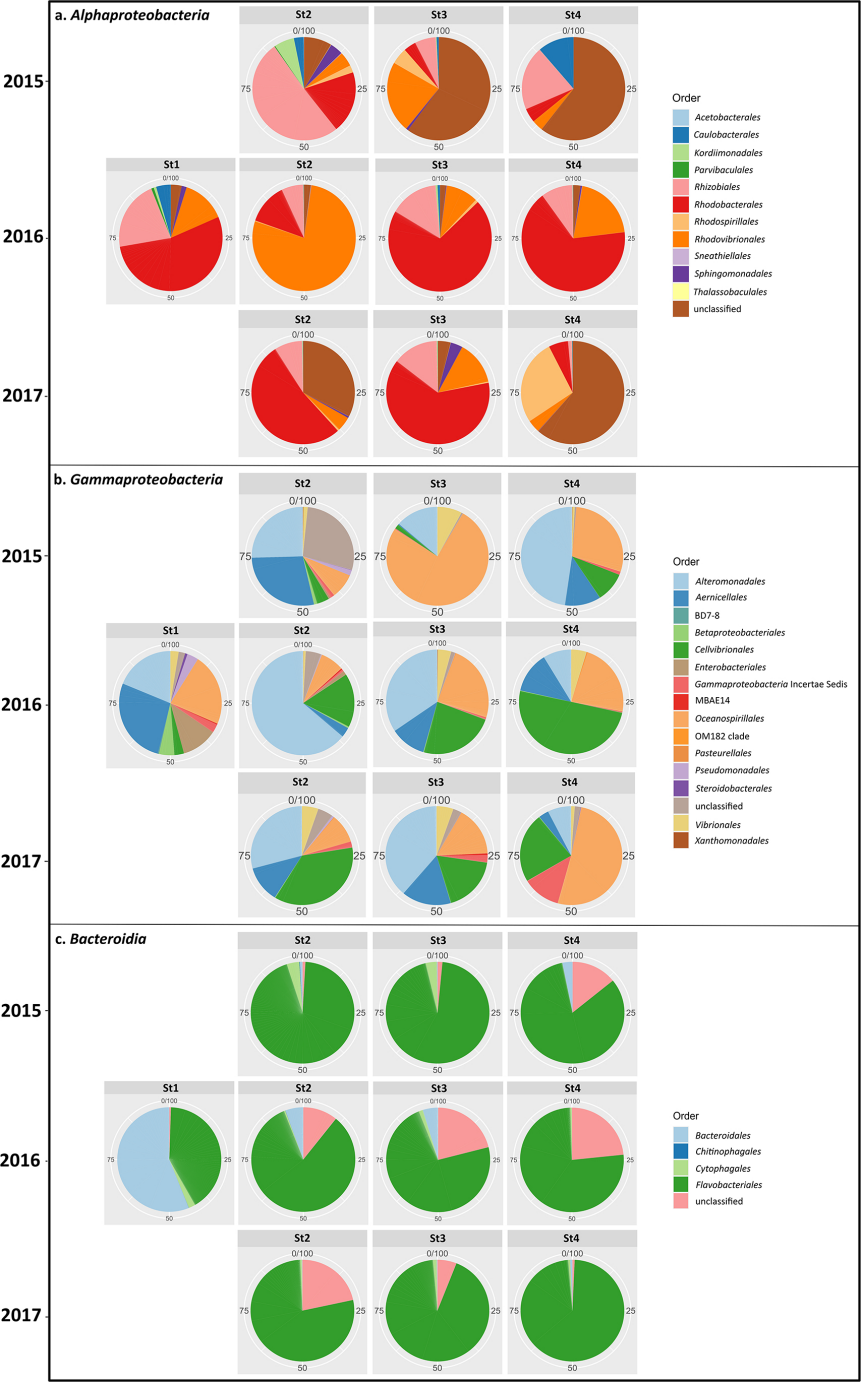
Relative abundance of bacterial orders in (a) *Alphaproteobacteria* and (b) *Gammaproteobacteria* and (c) the bacterial class *Bacteroidia*. There were more orders of *Alphaproteobacteria* and *Gammaproteobacteria* each year in earlier stages (stages 1 and 2). In *Bacteroidia*, *Flavobacteriales* was the dominant order.

We also investigated the dynamics of the three dominant classes—*Gammaproteobacteria*, *Alphaproteobacteria*, and *Bacteroidia*—across all maturation stages (Fig. 4a, b, and c). In *Alphaproteobacteria* and *Gammaproteobacteria*, bacteria were more diverse in earlier stages (stages 1 and 2); however, the order *Flavobacteriales* in *Bacteroidia* remained dominant throughout all stages.

**Fig. 5.**
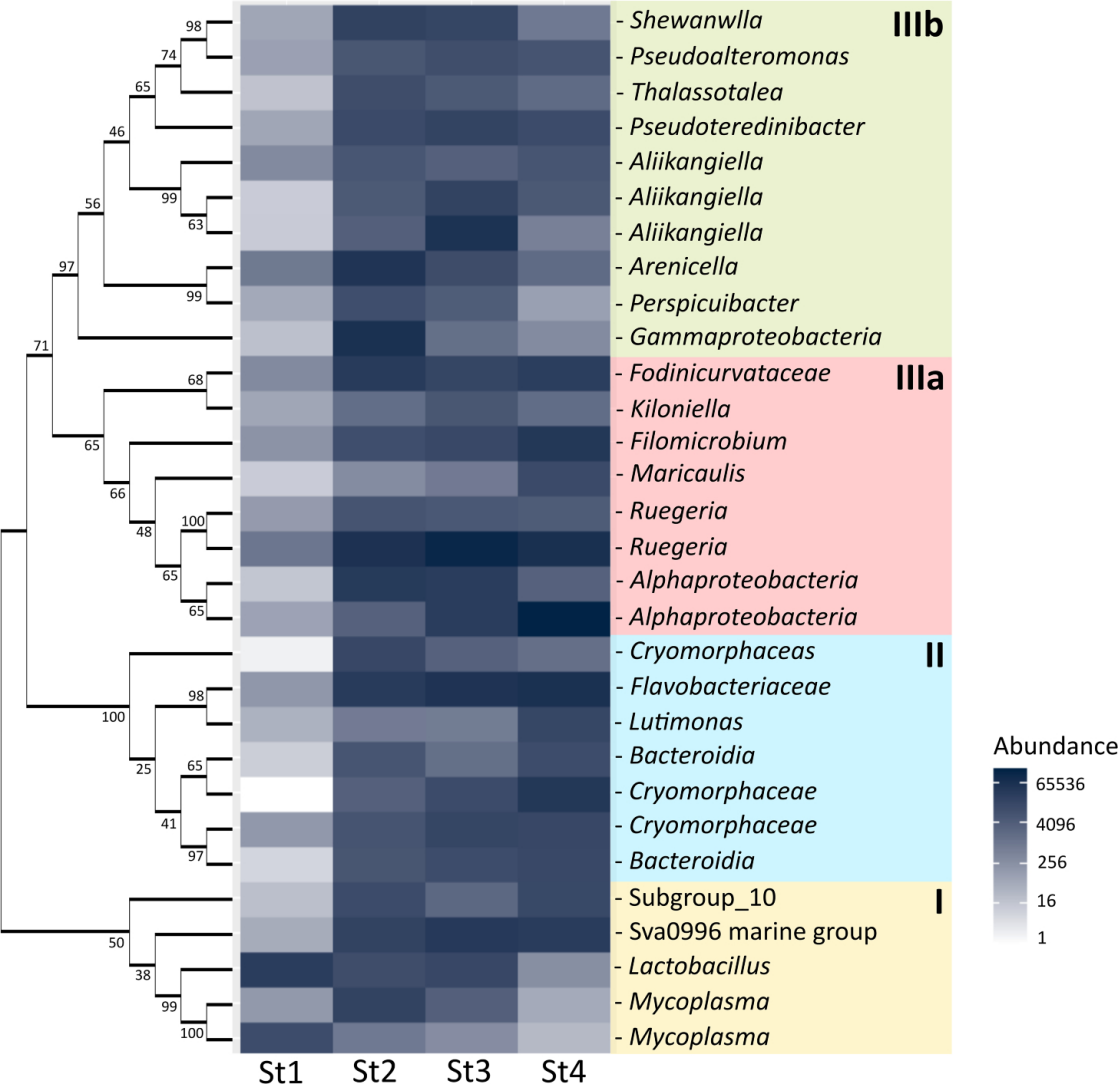
Heatmap of top 30 OTUs of shared 191 OTUs at different stages according to phylogenic relationships among 30 top OTUs. The first group (I) is *Mycoplasma* and Gram-positive bacteria. The second group (II) is *Bacteroidia*. The third group (III) is composed of *Alphaproteobacteria* (IIIa) and *Gammaproteobacteria* (IIIb).

There were 191 OTUs present in all stages, and each stage had multiple unique OTUs (Fig. S6). The earlier stages shared fewer OTUs with the late stages and more OTUs were shared among close stages. Stages 1 and 4 did not share any OTUs (Fig. S6). The core 191 OTUs may be resident bacteria and specific to the ANG. The differences observed in bacterial compositions across the years may be due to the environment (Fig. S4), which may also influence the core 191 OTUs. The abundance of the 191 bacterial OTUs also suggests a significant difference among the years (R^2^=0.126, *P*=0.01, Fig. S7). The heatmap plotted the top 30 of the core 191 OTUs at different stages, and clear shifts were observed within the three phylogeny groups (Fig. 5). Stage 1 was dominated by group I (Gram-positive), but not group II or III (Gram-negative). In group I, the abundance of *Mycoplasma*, *Lactobacillus*, and two other OTUs gradually decreased throughout the maturation process; however, this may have been natural succession. Group II contained classes of *Bacteroidetes*; *Bacteroidia* were abundant in stage 4, and the abundance of *Flavobacteriaceae* remained stable from stages 2 to 4. The dominant OTU (OTU12) in *Flavobacteriales* (Fig. S8) was the most closely related to the marine species *Pseudofulvibacter geojedonensis* (NR126234.1). Group III comprised *Alphaproteobacteria* (IIIa) and *Gammaproteobacteria* (IIIb), and their abundance shifted across the maturation stages—group IIIa gradually increased from stages 2 to 4, whereas IIIb showed the reverse.

**Fig. 6.**
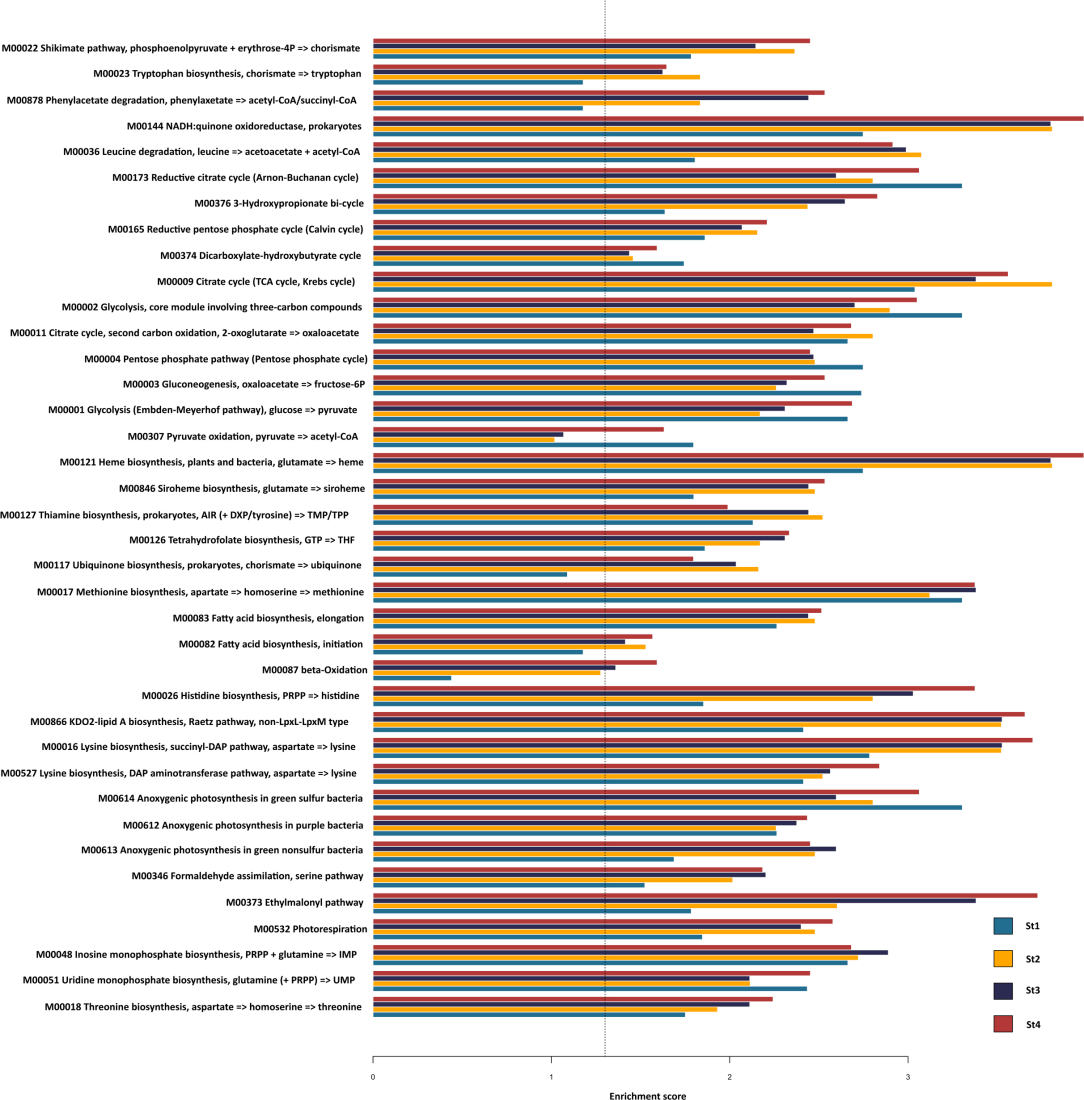
Enrichment analysis of predictive KEGG metabolic modules of total bacterial OTUs in different stages of *Sepioteuthis lessoniana* ANG. Thirty-eight modules were enriched in the microbiota of one or more of the ANG stages. The dashed line indicates that the FDR q-value=0.05.

After the PICRUSt2 functional prediction analysis, genes were categorized into KEGG metabolic modules. We found that NADH:quinone oxidoreductase, the citrate cycle, heme biosynthesis, KDO2-lipid A biosynthesis, and lysine biosynthesis had the highest scores (Fig. 6). The heme biosynthesis function had the highest score; it was low in stage 1 and increased throughout the maturation process. Functional predictions of the 191 core OTUs present in all stages showed that the heme biosynthesis function also had the highest score (Fig. S9). In addition, in the functional prediction of the dominant bacterial classes *Alphaproteobacteria* (Fig. S10), *Gammaproteobacteria* (Fig. S11), and *Bacteroidia* (Fig. S12), the heme biosynthesis function had a high score. Among these three classes, *Gammaproteobacteria* had the highest heme biosynthesis enrichment score (>3) in all stages, and *Gammaproteobacteria* and *Alphaproteobacteria* showed higher scores in stages 2 and 3. Although *Bacteroidia* had the lowest heme biosynthesis score, it increased with progressive ANG stages.

## Discussion

The microbial community in the ANG of S. *lessoniana* is influenced by the environment, the stage of maturity, and the host. The majority of studies on the ANG of mature cephalopods have considered the complex microbial consortium to be predominantly influenced by the environment. Due to a lack of information on microbial dynamics throughout the maturation process, it remains unclear whether the diversity of the complex microbial consortium remains similar in different mature stages. The present study incorporated spatial and temporal factors that may influence the dynamics of the microbial consortium in the ANG, which made it possible to identify the predominant driving factors and synergistic effects of the environment and maturity levels affecting the microbial composition. The results obtained suggest that the environment affects the microbial compositions of individuals, while maturity levels exert progressive effects on microbial dynamics over time.

*Alphaproteobacteria* are generally dominant in mature cephalopod ANG (Grigioni *et al.*, 2000; Barbieri *et al.*, 2001; Pichon *et al.*, 2005; Collins *et al.*, 2012; Kerwin and Nyholm, 2017). In the present study, *Alphaproteobacteria* were also dominant in mature ANG, similar to the findings of Kerwin and Nyholm (2017) and Collins *et al.* (2012), followed by *Gammaproteobacteria* and *Bacteroidia*. However, *Verrucomicrobia* were abundant in the ANG of the Hawaiian bobtail squid, *E. scolopes*, and the class *Opitutae* has been regarded as a core symbiotic group (Collins *et al.*, 2012; Kerwin and Nyholm, 2017). Although bacterial *Verrucomicrobia* were present in the early stages of some samples, they were not included in the top 30 suggested resident OTUs in *S. lessoniana*. The abundance of *Verrucomicrobia* in *E. scolopes* was consistent across locations and populations (Kerwin and Nyholm, 2017; 2018), indicating that they are species-specific symbionts. *E. scolopes* and *S. lessoniana* are from various orders and, thus, are expected to have different ANG core microbiomes. In *S. lessoniana*, we suggested that *Verrucomicrobia* are abundant in the earlier stage of ANG maturation, but are then replaced by other bacterial groups, such as *Bacteroidia*, *Alphaproteobacteria*, and *Gammaproteobacteria* in the mature stages.

In *E. scolopes*, the order *Flavobacteriales* in *Bacteroidia* was found at a consistent abundance of 10% (Collins *et al.*, 2012; Kerwin and Nyholm, 2017; 2018), which is in agreement with the present results on *S. lessoniana* across all maturation stages. Many members of the marine *Flavobacteriales* produce carotenoids—*e.g.*, *Algibacter* spp. (Park *et al.*, 2013), *Olley* spp. (Lee *et al.*, 2013), *Lacinutrix venerupis* (Lasa *et al.*, 2015), and *Mesoflavibacter zeaxanthinifaciens* (Asker *et al.*, 2007). Based on the phylogenetic results of *Bacteroidia*, the dominant OTU (OTU12) of *Flavobacteriales* was closely related to *P. geojedonensis*, which produces Flexirubin-type pigments (Yoon *et al.*, 2013).

Flexirubin is also a common pigment in some *Flavobacteriales* members, which produce carotenoids and form yellow-orange-colored bacterial colonies (Achenbach *et al.*, 1978)—*e.g.*, *Ichthyenterobacterium magnum* (Shakeela *et al.*, 2015), *Mesoflavibacter sabulilitoris* (Park *et al.*, 2014), and *Flavivirga amylovorans* (Yi *et al.*, 2012). During maturation, the cephalopod ANG turns red-orange and becomes darker; this has been attributed to ANG-associated *Alphaproteobacteria*, such as members of the family *Rhodobacteraceae* (Collins *et al.*, 2012). Based on the present results, we suggest that *Flavobacteriales* and *Alphaproteobacteria* both contribute to the pigmentation of the mature ANG of *S. lessoniana*.

One of the major functions of the ANG was suggested to be its provision of a bacteria consortium on the egg jelly coat (JC) (Kerwin and Nyholm, 2018) as an antimicrobial defense (Barbieri *et al.*, 1997; Kerwin *et al.*, 2019; Suria *et al.*, 2020). The high abundance of *Alphaproteobacteria* and *Gammaproteobacteria* found in the later stage of maturation in the present study may have had an antimicrobial function. Although the specific role of *Alphaproteobacteria* in the ANG of *S. lessoniana* remains unclear, the genus *Roseobacter* may produce antimicrobial compounds that inhibit other bacteria, such as *Vibrio fischeri* (Cude *et al.*, 2012). The *Gammaproteobacteria Pseudoalteromonas* isolated from the ANG and JC of the Hawaiian bobtail squid was also found to exhibit antibacterial activity (Suria *et al.*, 2020).

Bacteria diversity in the ANG may originate from the environment instead of parental transmission based on the variations observed in the bacterial community of *S. lessoniana* ANG in the present study over the three years of sampling. This may lead to not only different bacteria in different environments, but also different environments creating different selection pressures for cephalopods, which, in turn, may yield different ANG bacterial communities (Kerwin and Nyholm, 2018). Two distance populations of *E. scolopes* from Hawaii were found to have a subset of the bacterial community of the ANG characterized to one location; this may be due to differences in morphologies between the two host populations as well as the reef topology and biota diversity (Kerwin and Nyholm, 2018). Future studies are needed to identify the biotic and abiotic factors influencing the bacterial composition of the ANG.

The ANG microbial community shifts gradually throughout maturation, with diversity decreasing across the different stages of maturation. Microbial dynamics in other animal organs have different patterns. In the human gut, microbiome diversity increases with age (Radjabzadeh *et al.*, 2020). In the Hawaiian *E. scolopes*, the microbiome of its light organ has been shown to undergo a “reduction of complexity” as it matures (McFall-Ngai, 2014). Other studies on the ANG of various cephalopods, such as squids and cuttlefish, also reported that it harbors a complex microbial consortium (Collins *et al.*, 2012; Kerwin and Nyholm, 2017). In the case of *S. lessoniana*, this may be the result of host selection.

Li *et al.* (2019) indicated that the TF-like protein in the outer layer of the ANG played an important role in the selection of bacteria that are invaginated into the ANG of *S. lessoniana*. Even though the TF-like protein and lactotransferrin (LTF) may have antimicrobial functions in the host, both are considered to be essential for iron acquisition in Gram-negative bacteria. This may favor bacteria that acquire iron from their host using mechanisms such as heme and siderophores. Some strains of *Leisingera* (the *Roseobacter* clade) in the ANG of bobtail squids contain siderophore synthesis genes, which may help them acquire iron from their host’s environment (Collins *et al.*, 2015). The TF-like protein is dominantly expressed in the outer layer (epithelium cells) of the ANG (Li *et al.*, 2019), thereby facilitating the acquisition of iron by *Bacteroidia*, *Alphaproteobacteria*, and *Gammaproteobacteria*, which appear to be involved in the heme biosynthesis pathway. Furthermore, this protein may enhance divergence in bacterial compositions among more mature individuals (Fig. 2). In contrast, the TF-like protein stimulated a low iron environment in the outer layer of the ANG of *S. lessoniana*, which may, in turn, facilitate the use of manganese (Mn) by *Lactobacillus* for metabolism (Pandey *et al.*, 1994; Imbert and Blondeau, 1998). In the outer layer of the ANG, this iron-independent *Lactobacillus* may be involved in the selection of bacteria during the early stage of ANG development. Nevertheless, further studies are needed to establish whether the host limits iron outside the ANG or attempts to enrich it in order to promote the involvement of bacteria in heme production for the host.

In conclusion, the bigfin reef squid is one of the highest-valued fishery products in Asia; however, *S. lessoniana* trade still relies on catching the species wild in open water. Since coral reef destruction is increasing, spawning habitats have been decreasing in recent years, and, thus, aquaculture breeding techniques are now being used. These breeding methods have not yet created sufficiently high yields to reach the market volume. In contrast to other cephalopod model organisms, *S. lessoniana* has not yet been examined in detail, particularly the process of female sexual maturation and the effects of resident microbial communities. The present results revealed that the microbial community in the ANG changes throughout maturation, which may influence the host’s color and iron regulation mechanisms. These results will facilitate the development of future aquaculture systems in multiple ways; these systems may be improved by observing changes in the ANG-associated bacterial community in the laboratory or aquaculture systems. The identification of the selection pressures involved in the bacterial communities that *S. lessoniana* take into their ANG and elucidating the underlying mechanisms are important goals for future investigations.

## Citation

Yang, S.-H., Chen, C., Hsieh, Y. E., Yang, S.-Y., Li, H.-W., Ching, T.-Y., et al. (2021) Bacterial Dynamics in the Accessory Nidamental Gland of *Sepioteuthis lessoniana* throughout Maturation. *Microbes Environ ***36**: ME21030.

https://doi.org/10.1264/jsme2.ME21030

## Supplementary Material

Supplementary Material

## Figures and Tables

**Table 1.** Characteristics of sampled squids

Batch	Stage	Sampling date	ML (cm)	BW (g)	GW (g)	GSI (%)	Gonadal stage	Reproductive phase
1	2	Nov, 2015	15.5	216.5	0.4	0.2	PO	Immature female
1	2	Nov, 2015	17.0	195.7	0.5	0.2	PO	Immature female
1	2	Dec, 2015	23.5	774.5	0.7	0.1	PO	Immature female
1	3	Nov, 2015	26.0	938.3	1.3	0.1	PVO	Maturing female
1	3	Feb, 2016	22.0	611.8	1.0	0.2	PVO	Maturing female
1	3	Dec, 2015	25.0	818.3	2.7	0.3	PVO	Maturing female
1	4	Dec, 2015	26.0	986.8	>10	—	MO	Mature female
1	4	Feb, 2016	26.0	883.6	>10	—	MO	Mature female
1	4	Oct, 2015	21.5	522.2	>10	—	MO	Mature female
2	1	Sep, 2016	15.5	196.0	<0.1	—	PO	Juvenile female
2	1	Sep, 2016	14.5	211.4	<0.1	—	PO	Juvenile female
2	2	Oct, 2016	18.0	308.3	0.3	—	PO	Immature female
2	2	Oct, 2016	17.5	310.7	<0.1	—	PO	Immature female
2	2	Nov, 2016	26.0	912.0	0.6	0.1	PO	Immature female
2	3	Nov, 2016	23.0	717.0	0.8	0.1	PVO	Maturing female
2	3	Dec, 2016	n.d.	n.d.	n.d.	—	PVO	Maturing female
2	3	Dec, 2016	21.0	542.0	n.d.	—	PVO	Maturing female
2	3	Dec, 2016	n.d.	n.d.	n.d.	—	PVO	Maturing female
2	3	Feb, 2017	24.0	735.5	0.5	0.1	PVO	Maturing female
2	3	Feb, 2017	27.0	1120.0	0.7	0.1	PVO	Maturing female
2	4	Feb, 2017	25.5	991.0	>10	—	MO	Mature female
2	4	Mar, 2017	25.0	744.0	9.0	1.2	VO	Mature female
2	4	Mar, 2017	33.0	1787.0	>10	—	MO	Mature female
2	4	Mar, 2017	32.0	1650.0	>10	—	MO	Mature female
3	2	Nov, 2017	22.0	683.6	0.8	0.1	PO	Immature female
3	2	Dec, 2017	29.0	1321.8	1.9	0.1	PO	Immature female
3	2	Jan, 2018	23.5	680.8	1.4	0.2	PO	Immature female
3	2	Jan, 2018	27.5	977.7	1.7	0.2	PO	Immature female
3	3	Jan, 2018	28.7	1098.2	1.7	0.2	PVO	Maturing female
3	3	Jan, 2018	26.4	859.3	1.5	0.2	PVO	Maturing female
3	3	Jan, 2018	28.7	1252.9	1.7	0.1	PVO	Maturing female
3	3	Mar, 2018	24.7	695.1	1.3	0.2	PVO	Maturing female
3	4	Nov, 2017	29.0	1214.3	>10	—	MO	Mature female
3	4	Dec, 2017	31.0	1265.7	4.9	0.4	VO	Mature female
3	4	Jan, 2018	23.5	891.5	>10	—	MO	Mature female
3	4	Mar, 2018	26.5	1039.6	>10	—	MO	Mature female
