# Supplementary material for: Bacterial Dynamics in the Accessory Nidamental Gland of Sepioteuthis lessoniana throughout Maturation
Source: Microbes Environ. 2021 Oct 2;36(4):ME21030. doi: 10.1264/jsme2.ME21030 (PMC8674444; doi:10.1264/jsme2.ME21030)

Supporting Information

Fig. S1 The squid sampling area. (a) The northeast coast of Taiwan. (b) The squids were collected by hand jigging on boat in the area shown in grey.

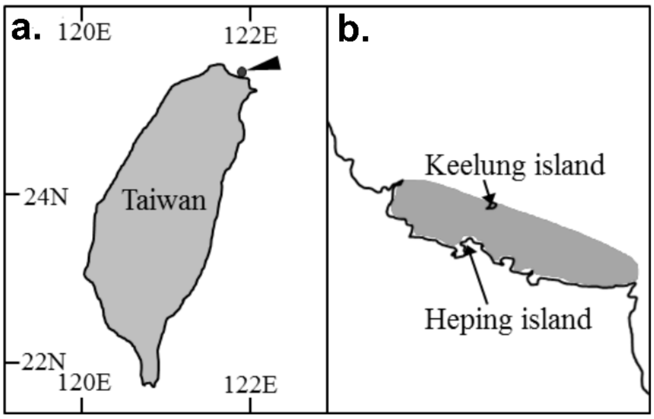

Fig. S2 Rarefaction curve of bacterial richness at different stages of *Sepioteuthis lessoniana*'s ANG in different years. Years 2015, 2016 and 2017 correspond to batches 1, 2, and 3, respectively.

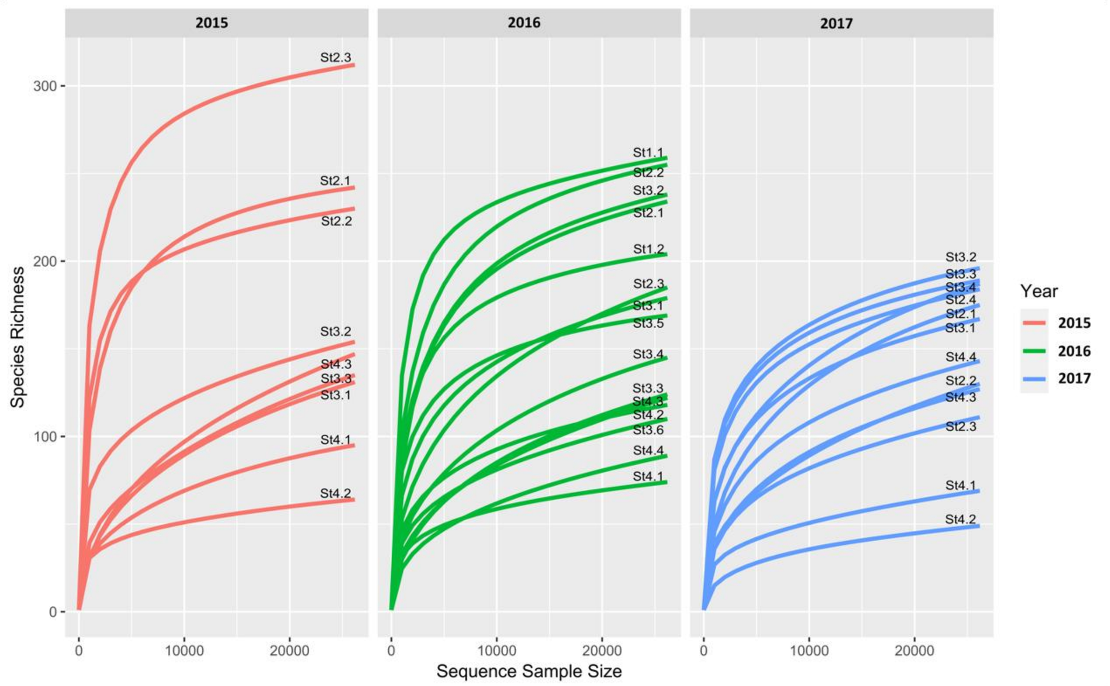

Fig. S3 The bacterial diversity indices of *Sepioteuthis lessoniana*'s ANG from different sampling years. Years 2015, 2016 and 2017 correspond to batches 1, 2, and 3, respectively.

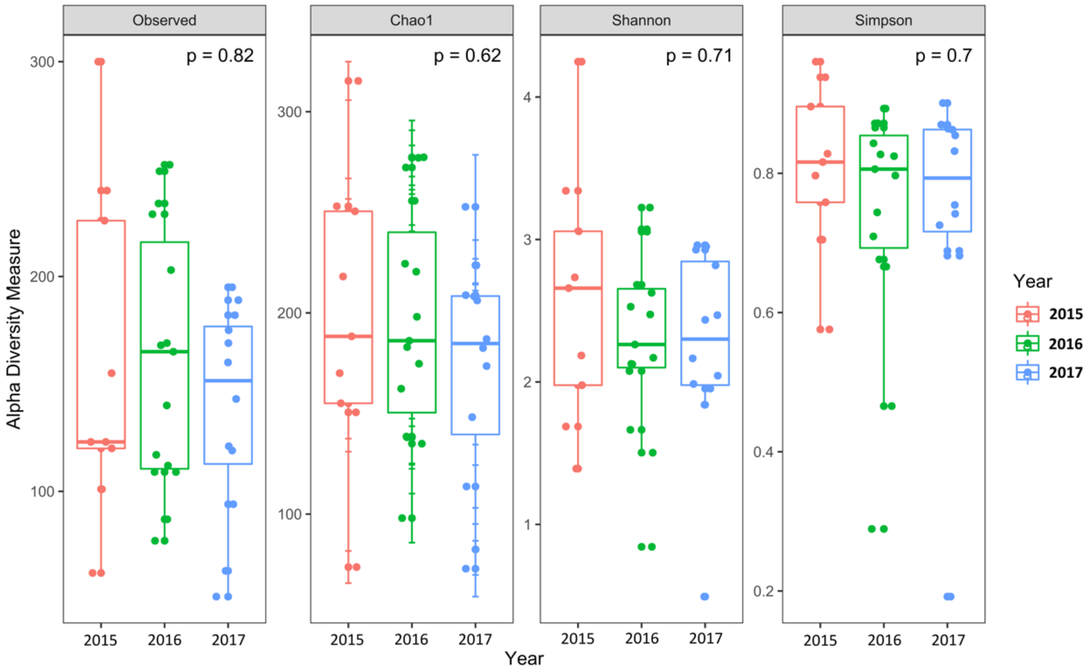

Fig. S4 The Principal Coordinate Analysis (PCoA) of bacterial OTUs from different years. Years 2015, 2016 and 2017 correspond to batches 1, 2, and 3, respectively.

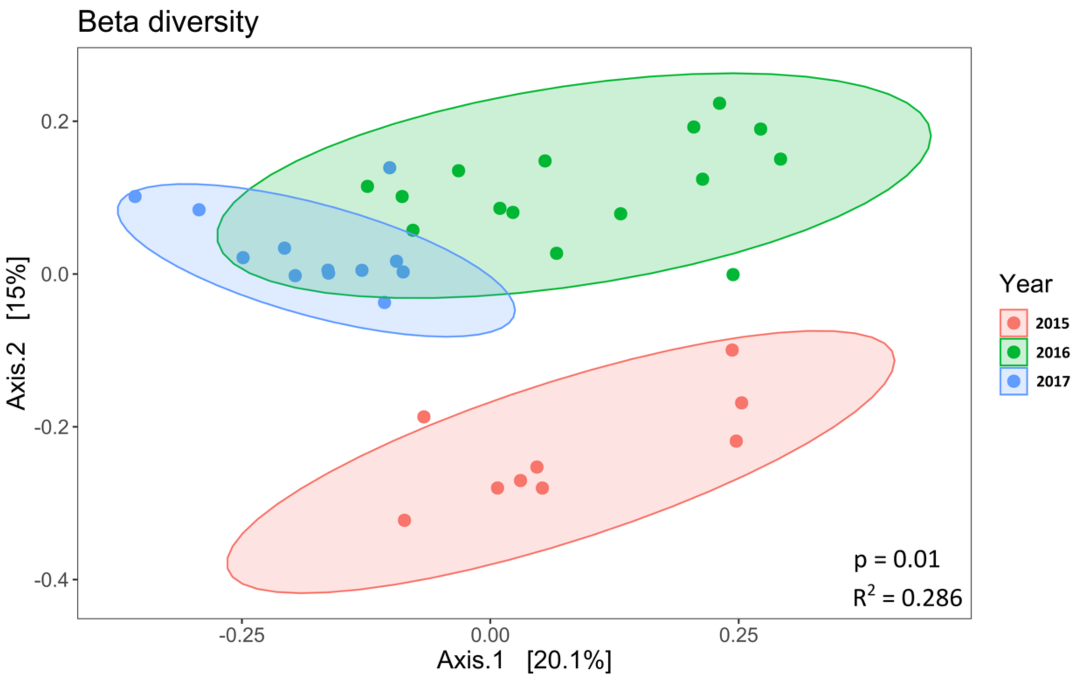

Fig. S5 The bacterial diversity indices of *Sepioteuthis lessoniana*'s ANG in different stages.

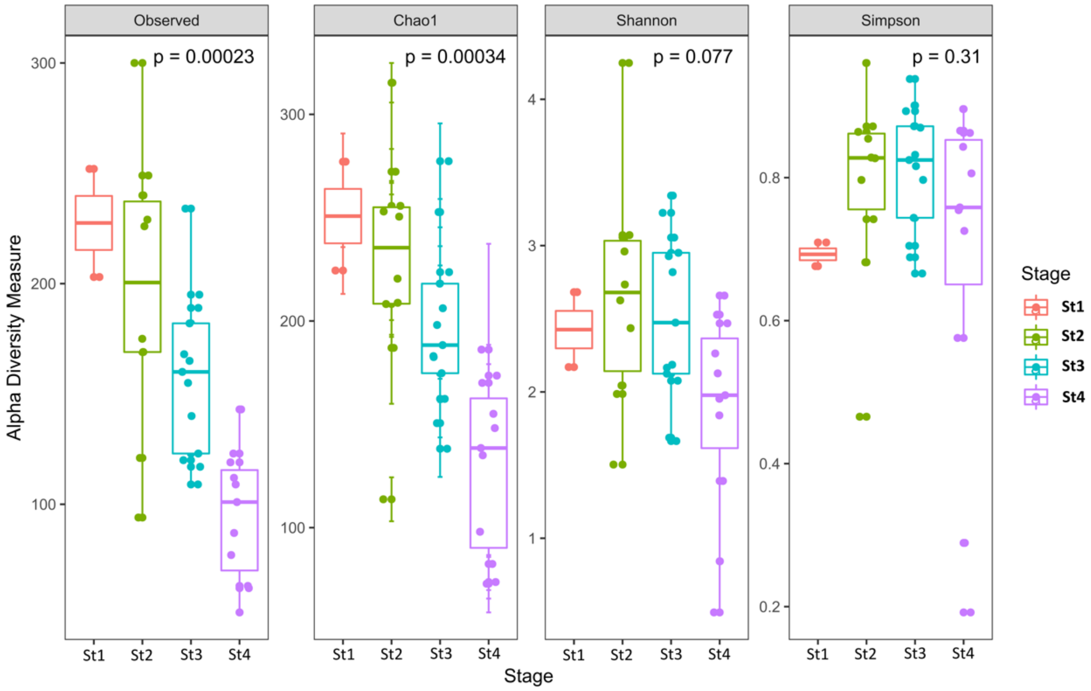

Fig. S6 Venn diagram of *Sepioteuthis lessoniana*-associated bacterial OTUs in different stages of ANG. 191 OTUs were found in all of the stages.

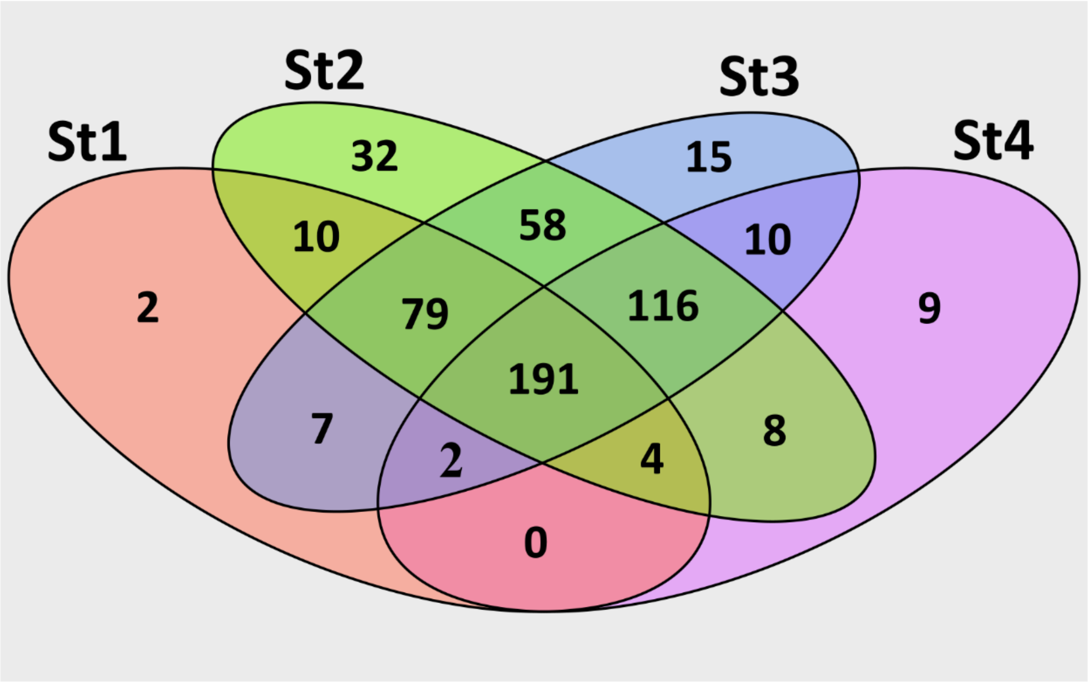

Fig. S7 The PCoA of the 191 potential residential bacterial OTUs in different years.

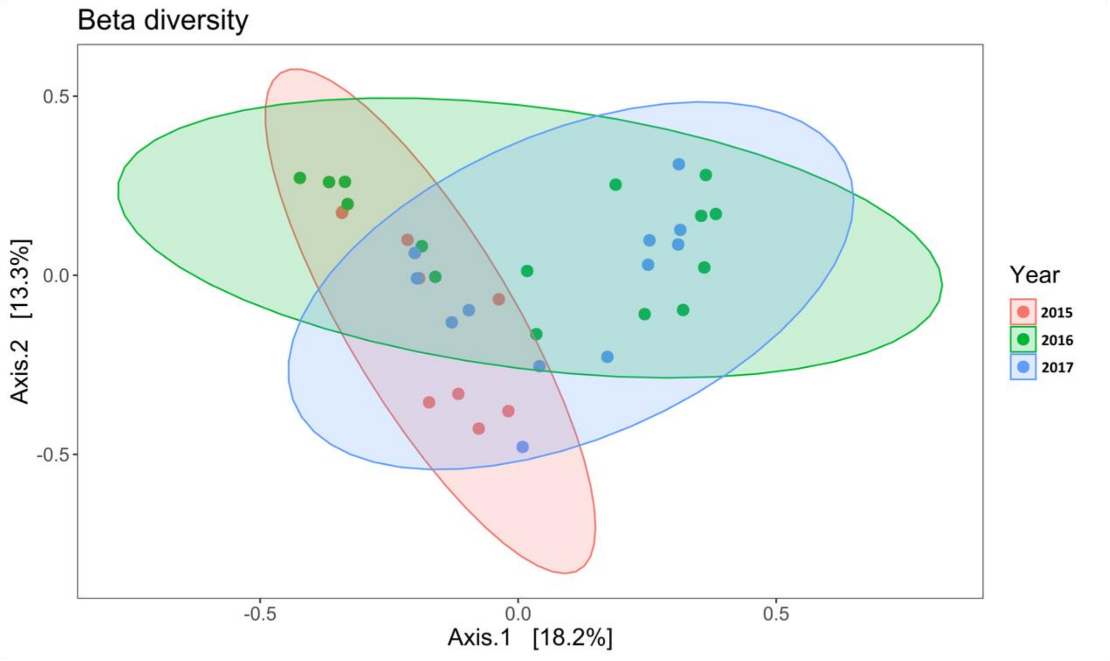

Fig. S8 Maximum Likelihood tree of OTU12 and Flavobacteriaceae. The species most closely related to OTU12 is *Pseudofulvibacter geojedonensis*, which was isolated from a marine environment and was able to produce the Flexirubin-like pigment. The replication number for Bootstrapping analysis is 1000.

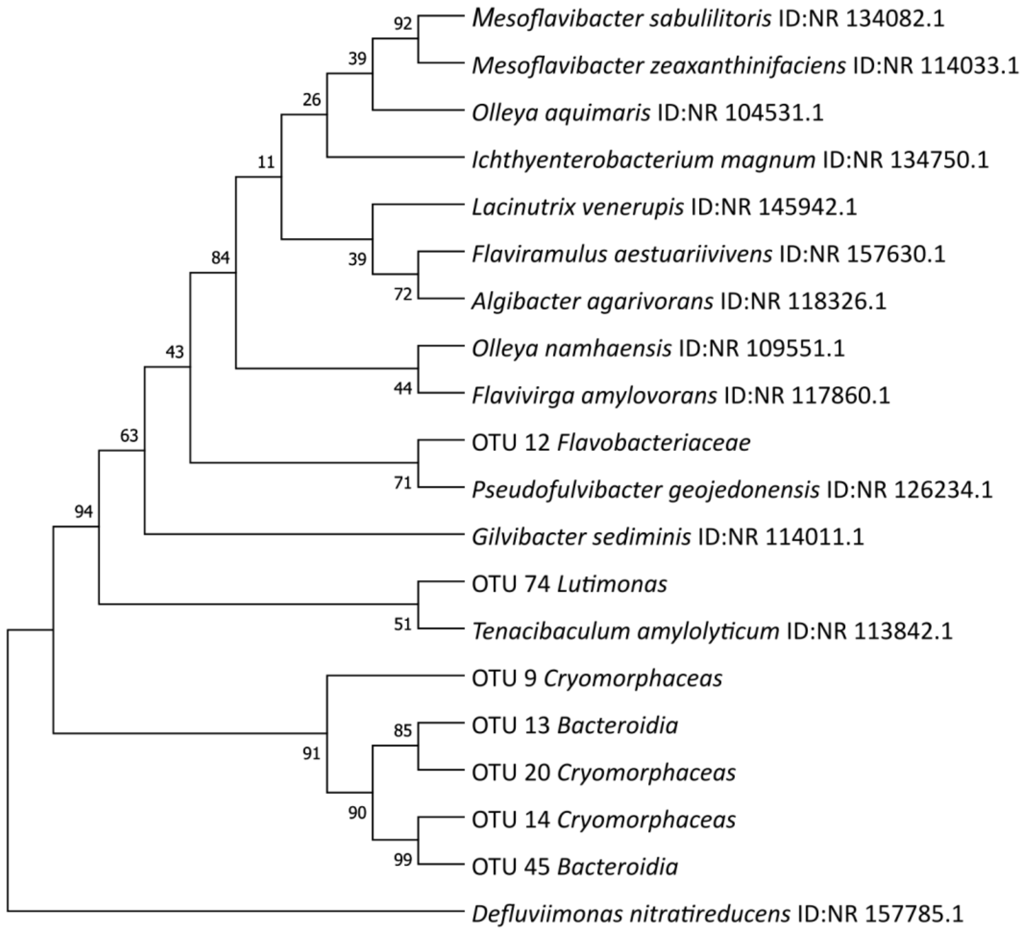

Fig. S9 Enrichment analysis for predictive KEGG metabolic modules of 191 core bacterial OTUs in different stages of *S. lessoniana* ANG. Thirty-five modules were enriched in the microbiota of one or more of the ANG stages. The dashed line indicates that the FDR q-value=0.05.

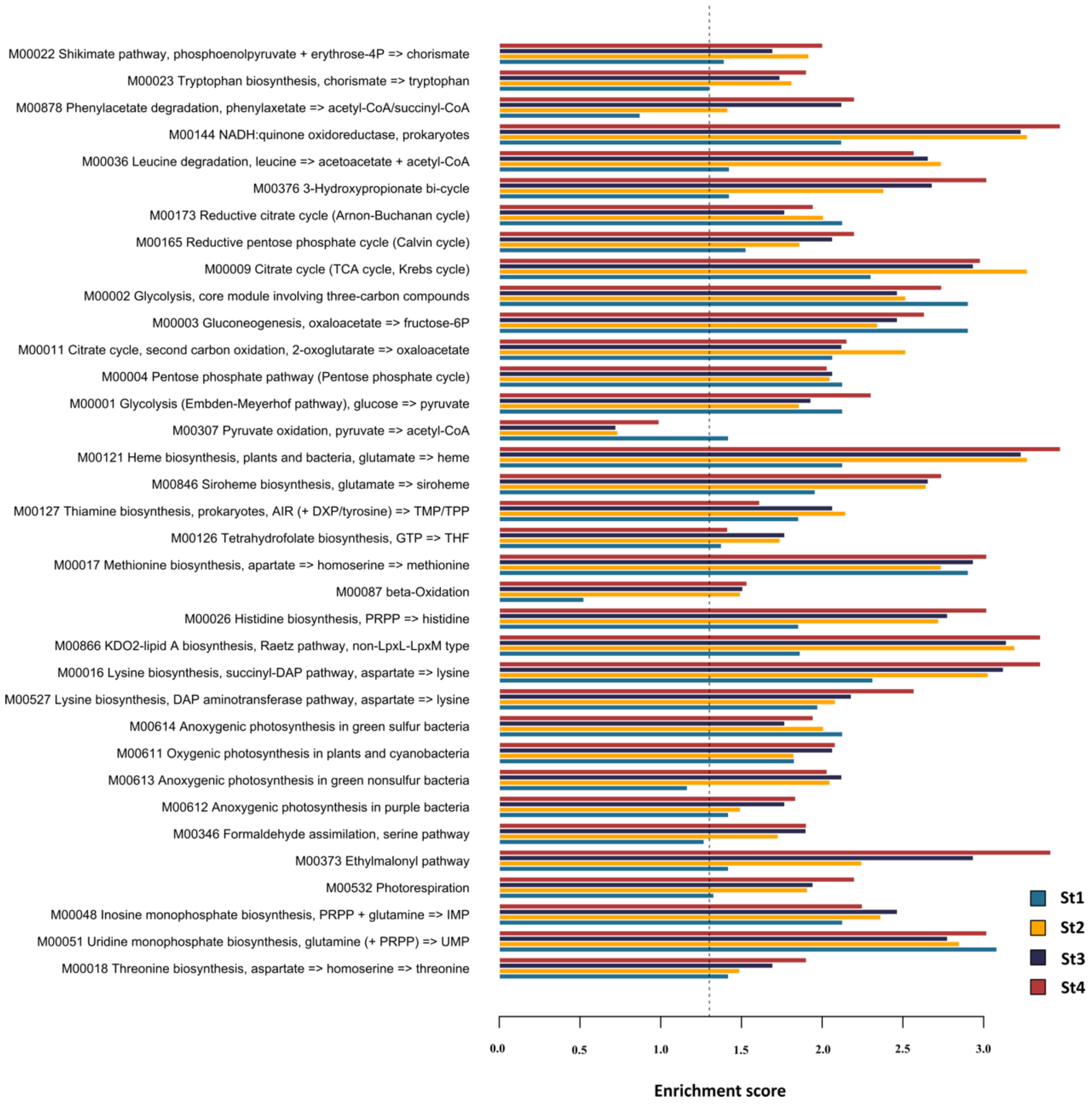

Fig. S10 Enrichment analysis for predictive KEGG metabolic modules of OTUs belonging to class *Alphaproteobacteria* in different stages of *S. lessoniana* ANG. Thirteen modules were enriched in the microbiota of one or more of the ANG stages. The dashed line indicates that the FDR q-value=0.05.

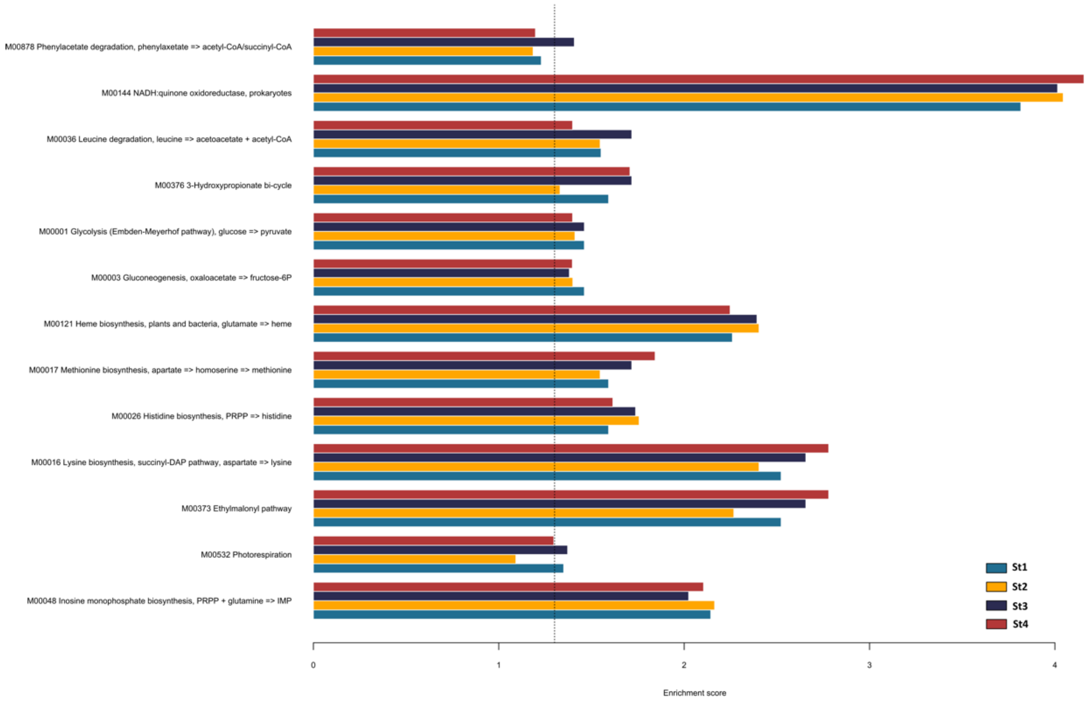

Fig. S11 Enrichment analysis for predictive KEGG metabolic modules of OTUs belong to class *Gammaproteobacteria* in different stages of *S. lessoniana* ANG. Twenty-one modules were enriched in the microbiota of one or more of the ANG stages. The dashed line indicates that the FDR q-value=0.05.

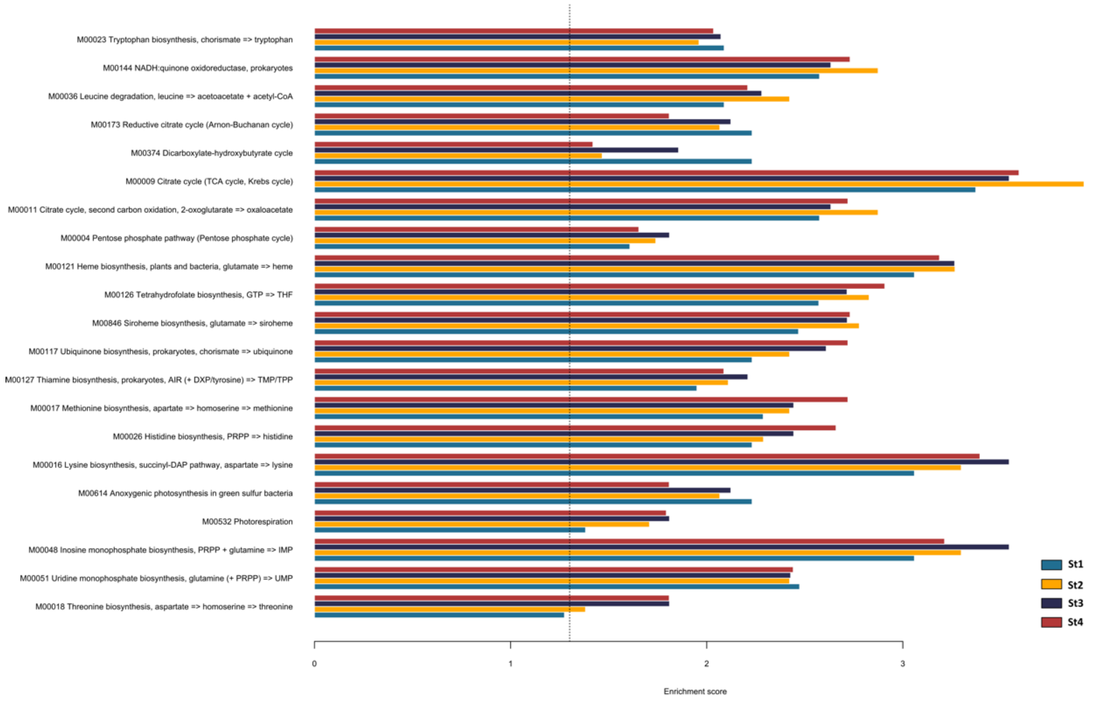

Fig. S12 Enrichment analysis for predictive KEGG metabolic modules of OTUs belong to class *Bacteroidia* in different stages of *S. lessoniana* ANG. Ten modules were enriched in the microbiota of one or more of the ANG stages. The dashed line indicates that the FDR q-value=0.05.

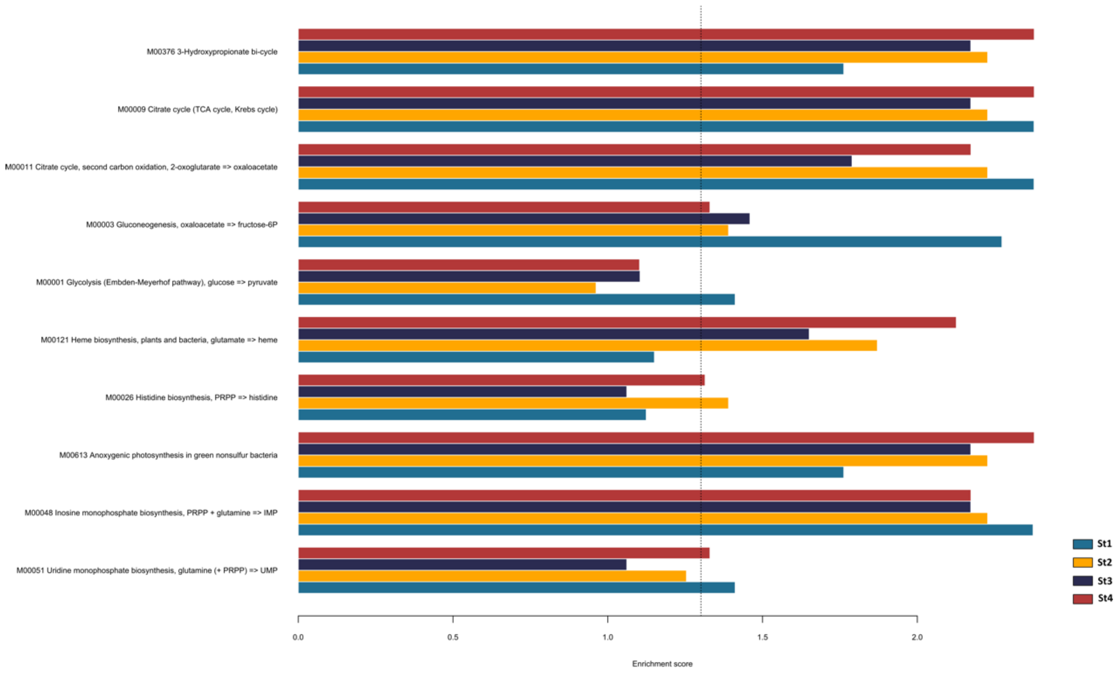

Supplement: Supplementary file 1 — Supplementary Material [file 36_21030_s1.pdf]
